# *PIK3CA* mutation correlates with mTOR pathway expression but not clinical and pathological features in Fibfibroipose vascular anomaly (FAVA)

**DOI:** 10.1186/s13000-022-01199-3

**Published:** 2022-01-30

**Authors:** Yumiko Hori, Katsutoshi Hirose, Michio Ozeki, Kenji Hata, Daisuke Motooka, Shinichiro Tahara, Takahiro Matsui, Masaharu Kohara, Hiroki Higashihara, Yusuke Ono, Kaishu Tanaka, Satoru Toyosawa, Eiichi Morii

**Affiliations:** 1 Department of Pathology, Osaka University Graduate School of Medicine, 2-2 Yamadaoka, 565-0871 Suita, Osaka Japan; 2 Department of Central Laboratory and Surgical Pathology, National Hospital Organization, Osaka National Hospital, 2-1-14 Hoenzaka, Chuo-ku, Osaka-shi, 540-0006 Osaka Japan; 3 Department of Oral Pathology, Osaka University Graduate School of Dentistry, 1-8 Yamadaoka, 565-0871 Suita, Osaka Japan; 4 Department of Pediatrics, Graduate School of Medicine, Gifu University, 1-1 Yanagido, 501-1194 Gifu, Japan; 5 Department of Molecular and Cellular Biochemistry, Osaka University Graduate School of Dentistry, 1-8 Yamadaoka, 565-0871 Suita, Osaka Japan; 6 Genome Information Research Center, Research Institute for Microbial Diseases, Osaka University, 3-1 Yamadaoka, 565-0871 Suita, Osaka Japan; 7 Department of Radiology, Osaka University Graduate School of Medicine, 2-2 Yamadaoka, 565-0871 Suita, Osaka Japan

**Keywords:** Fibro-adipose vascular anomaly, FAVA, PIK3CA, mTOR, Vascular anomaly, Lymphatic malformation, Venous malformation, Sirolimus

## Abstract

**Background:**

Fibro-adipose vascular anomaly (FAVA) is a rare and new entity of vascular anomaly. Activating mutations in the *phosphatidylinositol-4,5-bisphosphate 3-kinase catalytic subunit alpha* (*PIK3CA*) gene were identified at a frequency of 62.5% in FAVA cases. The *PIK3CA* mutations excessively activate mammalian target of rapamycin (mTOR) pathway, which promotes angiogenesis and lymphangiogenesis, implying tha*t PIK3CA* mutations may act as drivers of FAVAs. This study investigated the correlations between *PIK3CA* mutational status, clinicopathological features and immunohistochemical expression of the mTOR pathway in a series of FAVA.

**Methods:**

We retrospectively evaluated the clinical and pathological findings of four FAVA cases. We performed next-generation sequencing (NGS) with a custom panel of genes associated with the mTOR pathway and genes responsible for other vascular anomalies; followed by direct sequencing and immunohistochemical analysis of the mTOR pathway.

**Results:**

Two PIK3CA-mutation cases and two PIK3CA-wild-type (wt) cases exhibited similar typical clinical features of FAVA. Histological analysis revealed venous malformation, lymphatic malformation, nerves containing enlarged abnormal vessels and fibrofatty tissue were observed regardless of *PIK3CA* mutational status. In contrast to clinical and histological findings, the immunohistochemical expression of activated AKT and mTOR that are upstream of the mTOR pathway was detected in abnormal vessels of PIK3CA-mutation cases but not in those of PIK3CA-wt cases. However, activated eukaryotic translation initiation factor 4E-binding protein 1 (4EBP1) and ribosomal protein S6 kinase 1 (S6K1), both of which are downstream effectors of the mTOR pathway, were expressed in abnormal vessels of both PIK3CA-mutation and PIK3CA-wt cases. Furthermore, targeting NGS did not find any common genetic mutations involved in the mTOR pathway among PIK3CA-wt cases.

**Conclusions:**

There was no significant association between the presence of *PIK3CA* mutations and the clinicopathological features of FAVA, suggesting that the *PIK3CA* gene is not necessarily involved in the onset of FAVA. FAVAs lacking *PIK3CA* mutations may be caused by other gene mutations that activate 4EBP1 and S6K1.

## Background

Fibro-adipose vascular anomaly (FAVA) is a newly described vascular anomaly [1]. FAVA is extremely rare and occurs most commonly in the muscles of the lower extremities of young patients [1, 2]. Histologically, FAVA is composed of venous malformation (VM), lymphatic malformation (LM), and the presence of fibro-adipose tissues with the atrophic skeletal muscle [1, 3]. A recent study identified somatic and mosaic gain-of-function mutations of the *phosphatidylinositol-4,5-bisphosphate 3-kinase catalytic subunit alpha* (*PIK3CA*) gene in a subset of FAVAs (62.5%) [4]. The identified *PIK3CA* mutations are p.E542K, p.E545K and p.Q546K in the helical domain (encoded within exon 9), and p.H1047R in the kinase domain (encoded within exon 20) [4]. These *PIK3CA* mutations are termed hotspot mutations, and are present in a subset of VMs and the majority of LMs [4–10]. *PIK3CA* mutations excessively activate the phosphoinositide 3-kinase (PI3K)/AKT/mammalian target of rapamycin (mTOR) pathway in the endothelial cells during vascular developments [4–10]. Activation of PI3K results in the phosphorylation of AKT (p-AKT), and p-AKT phosphorylates mTOR. Furthermore, the phosphorylated form of mTOR (p-mTOR) phosphorylates downstream effectors such as eukaryotic translation initiation factor 4E-binding protein 1 (4EBP1) and ribosomal protein S6 kinase 1 (S6K1), ultimately promoting angiogenesis and lymphangiogenesis [11, 12]. These results highly suggested that *PIK3CA* mutations may act as drivers of FAVAs through activation of the PI3K/AKT/mTOR pathway. Furthermore, the presence of *PIK3CA* mutations in VMs and the genotype of *PIK3CA* mutation in LMs correlate with both clinical severity and histological features [8, 10, 13]. However, little is known regarding the correlations among *PIK3CA* mutational status, the mTOR pathway activation status and clinicopathological features in FAVA. Here, we report the results of clinical, histological, immunohistochemical, and genetic analyses examining a small series of isolated FAVA cases.

## Methods

Four FAVA cases with formalin-fixed paraffin-embedded (FFPE) tissues were retrieved from the pathology files of Osaka University Hospital from 2010 to 2020. A final diagnosis of FAVA was determined by consensus agreement after consideration of clinical, radiologic, and histological findings [1–3]. This study was approved by the Ethical Review Board of the Graduate School of Medicine, Osaka University (IBR No. 17,214).

### Next-generation sequencing (NGS)

Genomic DNA was extracted from FFPE tissue using the QIAamp DNA FFPE Tissue Kit (Qiagen, Valencia, CA, USA) according to the manufacturer’s instructions. Two pathologists (Y.H. and K.H.) selected FFPE blocks with greater than 50% abnormal tissue content in all cases. The gene panel was designed by SureDesign (https://earray.chem.agilent.com/suredesign) to cover a whole exon of 14 genes associated with the mTOR pathway signaling or responsible for other vascular anomalies (*PIK3CA*, *TEK*, *GNA11*, *GNAQ*, *AKT1*, *PTEN*, *mTOR*, *CCM*, *BRAF*, *MAP3K3*, *KRAS*, *NRAS*, *HRAS*, *RASA1*). On average 70 ng of the extracted DNA was fragmented by SureSelect Fragmentation Enzyme (Agilent Technologies, Inc. Santa Clara, CA, USA ) to 150–200 bp. Sequence libraries were prepared with a custom SureSelect Low Input Target Enrichment System (Agilent Technologies, Inc. Santa Clara, CA, USA) according to the manufacturer’s instructions and sequenced with the Illumina MiSeq (Illumina, San Diego, CA, USA). SureCall ver4.0 (https://www.agilent.com/en/download-software-surecall) was used for variant calling. DNA in introns or non-cording DNA were excluded. To confirm *PIK3CA* gene mutations, polymerase chain reaction (PCR) assays and direct sequencing were performed using the following primers: PIK3CA-Exon9 Forward, CAGCTCAAAGCAATTTCTAC; PIK3CA-Exon9 Reverse, CACTTACCTGTGACTCCAT; PIK3CA-Exon20 Forward, AACTGAGCAAGAGGCTTTGG; PIK3CA-Exon20 Reverse, TGTGTGGAAGATCCAATCCA. A mixture of 5% PIK3CA-mutant DNA against a background of 95% wild-type (wt) DNA was used as a positive control.

### Histological and immunohistochemical staining

Resected tissue samples were fixed with 10% formalin, routinely embedded in paraffin, cut into 4 μm thick serial sections, and used for H&E and immunohistochemical staining. Immunohistochemical staining was performed using a Roche Ventana BenchMark GX autostainer (Ventana Medical Systems, Tucson, AZ, USA) according to the manufacturer’s instructions. Primary antibodies against p-AKT (#4060, 1:100; Cell Signaling Technology, Danvers, MA, USA), p-mTOR (clone 49F9, 1:100; Cell Signaling Technology), p-S6K1 (#9204, 1:100; Cell Signaling Technology), p-4EBP1 (clone 236B4, 1:500; Cell Signaling Technology), S100 (polyclonal, Ventana Medical Systems), CD31 (clone JC70A, 1:200, Dako), CD34 (clone QBEnd10, 1:200, Dako), D2-40 (760-4395, Ventana Medical System), and PROX1 (ab199359, 1:500; Abcam, Cambridge, UK) were used. Samples were considered positive when at least 10% of the endothelial cells of abnormal vessels exhibited a signal for the targeted protein.

## Results

### Clinical data and molecular genetic findings

**Fig. 1:**
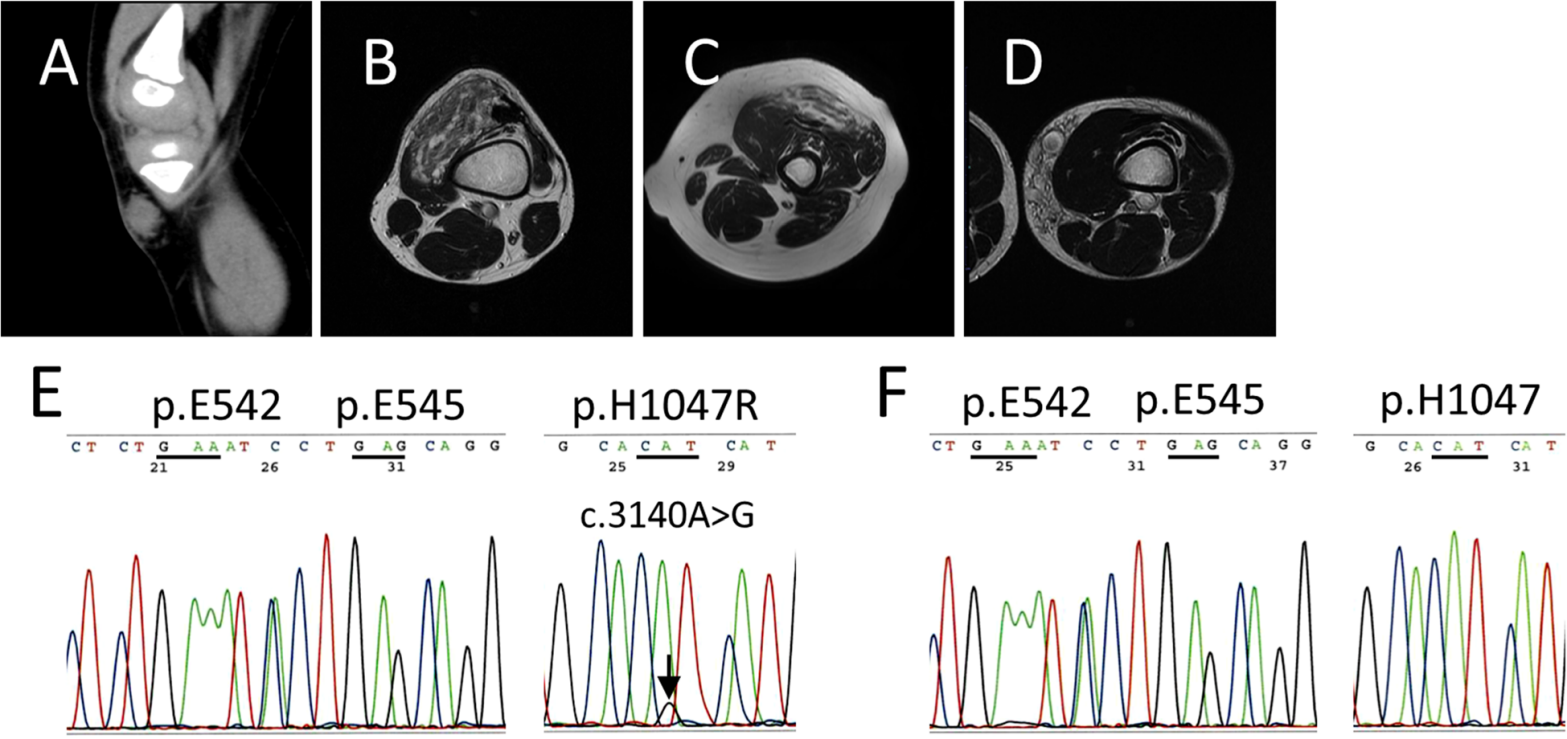
** A-D** Computed Tomography (CT) or magnetic resonance image (MRIs) analysis. Sagittal CT image of case 1 (**A**), and axial T2-weighted MRI of case 2 (**B**), case 3 (**C**) and case 4 (**D**). **E, F** DNA sanger sequencing of hotspot mutations in *PIK3CA.* Sequencing of *PIK3CA* for each FAVA cases showing chromatograms for c.3140 A>G (p.H1047R) (**E**) or wild-type (**F**)

The four patients included four men, and they ranged in age from 3 to 15 years (median, 12.5 years). Two patients presented at birth (cases 1 and 3). The presenting symptoms were pain (4/4 cases), swelling (3/4 cases), and functional restriction (2/4 cases). The preoperative clinical diagnosis was vascular anomalies, including infantile hemangioma, vascular malformation, and FAVA. The lesions were located within and in the vicinity of the thigh muscles, and knee (Fig. 1 A-D). Heterogenous *PIK3CA* hotspot mutation (p.H1047R) was identified in two cases (cases 1 and 2) (Fig. 1E), while none of the *PIK3CA* mutations were detected in other two cases (cases 3 and 4) (Fig. 1 F). We also found mutations in *TEK* in 2 cases, *GNA11* in 1 case, *AKT1* in 1 case, *PTEN* in 2 cases and *HRAS* in 1 case. The clinical characteristics and the results of the genetic analysis are summarized in Table 1.

**Table 1:** Clinical and molecular genetic summary of the patients

Case	Age/Sex	Location	Duration	Clinical diagnosis	Symptom	*PIK3CA* mutation	Other mutations
1	3/M	Knee	3 years	Infantile hemangioma	Pain, increased swelling	p.H1047R	*PTEN* (c.63dupG)
2	15/M	Thigh muscle	3 months (After Sclerotherapy)	Vascular malformation	Pain, functional restriction, increased swelling,	p.H1047R	*TEK* (p.W189*), *GNA11* (p.E191K), *AKT1* (p.R15Q, p.Y229=), *PTEN* (c.63dupG), *HRAS* (p.R102W)
3	13/M	Thigh muscle	13 years	Venous malformation	Pain, increased swelling	No mutation	No mutation
4	12/M	Thigh muscle	6 months	Venous malformation or FAVA	Pain, functional restriction	No mutation	*TEK* (p.R842H)

*PIK3CA*; *phosphatidylinositol-4,5-Bisphosphate 3-Kinase Catalytic Subunit Alpha, TEK*; *TEK receptor tyrosine kinase, AKT1*; *AKT serine/threonine kinase 1, PTEN*; *phosphatase and tensin homolog, HRAS*; *HRas proto-oncogene, GTPase*

### Histological and immunohistochemical findings

**Fig. 2:**
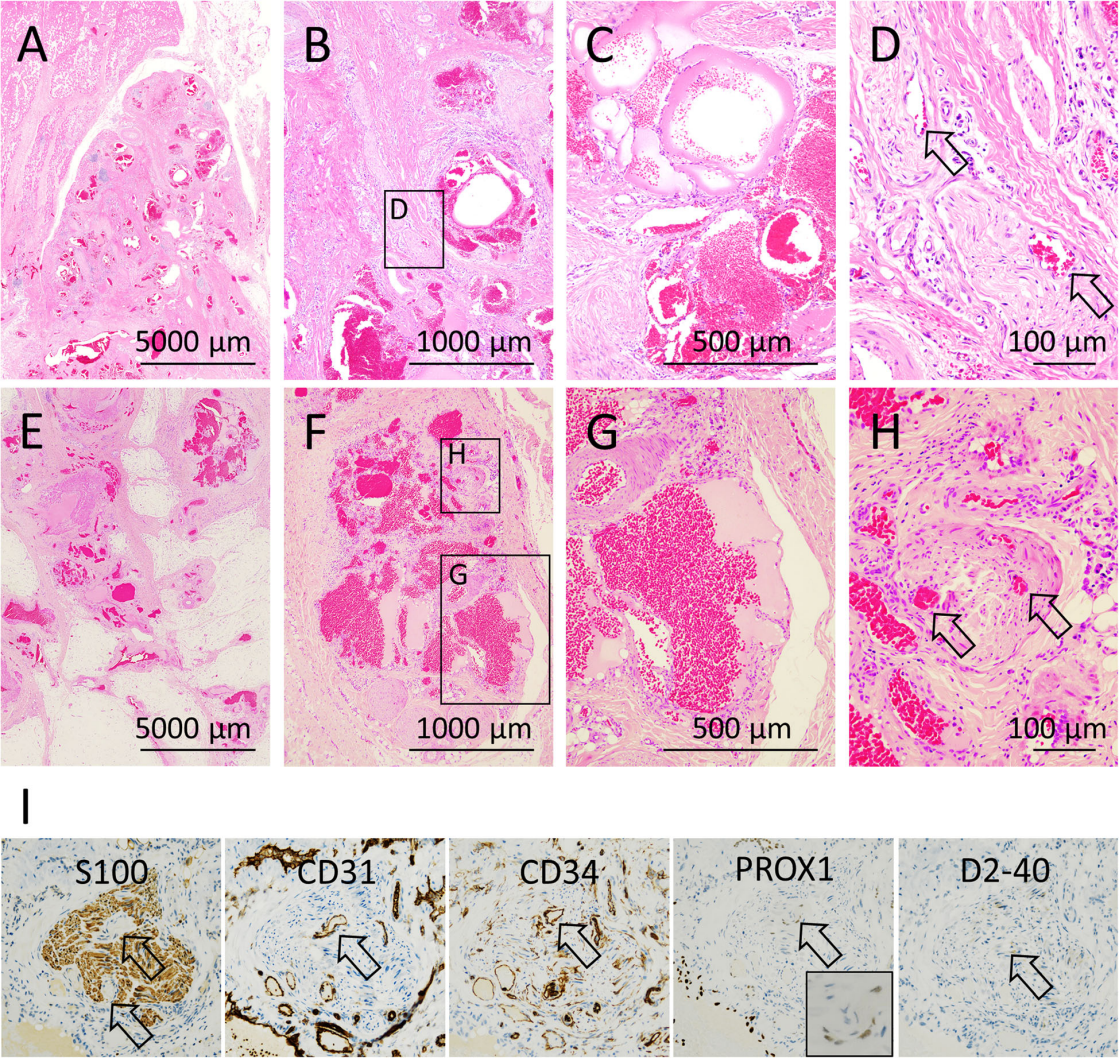
Histology and immunohistochemical analysis of FAVA. **A-H** Representative histological findings in PIK3CA-mutation case (**A-D**) and PIK3CA-wild type (wt) case (**E-H**). Vascular malformation and adipose and dense fibrous tissue (**A, E**; lower magnification and **B, F**; higher magnification). Clusters of vascular channels with thin-walled back-to-back blood-filled sacs (**C, G**). Nerve containing enlarged vessels (**D, H** arrows). **I** Serial sections of a nerve containing enlarged vessels (**H**) stained for S100, CD31, CD34, PROX1, and D20-40 **(**arrows indicated abnormal vessels in nerve)

The PIK3CA-mutation cases (cases 1 and 2) (Fig. 2 A-D) and PIK3CA-wt cases (cases 3 and 4) (Fig. 2E-H) possessed similar histology. All four cases exhibited abnormal vessels surrounded by dense fibrous tissue and adipose tissue with atrophic skeletal muscle (Fig. 2 A, E). The abnormal vessels were composed of VM and LM (Fig. 2B, F). The majority of the LM components possessed vascular clusters consisting of thin-walled back-to-back blood-filled sacs (Fig. 2 C, G). The lymphatic phenotype was supported by endothelial D2-40 and/or Prox1 immunopositivity in consistent with our previous study [3]. In one PIK3CA-mutant case (case 2) and one PIK3CA-wt case (case 3), some nerves contained enlarged vessels (Fig. 2D, H). The endothelial cells of these vessels within nerves were positive for CD31 (marker for endothelial cells) and CD34 (marker for blood vessels), negative to weakly positive for PROX1 (marker for lymphatic vessels), and negative for D2-40 (marker for lymphatic vessels) (Fig. 2I). That is, the vessels within nerves had the vein-like characteristics. The other findings included the observation of organized thrombi within abnormal veins in two cases (cases 2 and 4) and lymphocytic aggregates surrounding abnormal vessels in three cases (cases 2-4). The histological findings of all cases are summarized in Table 2.

**Table 2:** Summary of histological findings

Case	VM	LM	Nerve containing enlarged abnormal vessels	Organized thrombi	Lymphocytic aggregates
1	+	+	-	-	-
2	+	+	+	+	+
3	+	+	+	-	+
4	+	+	-	+	+

VM; venous malformation, LM; lymphatic malformation

**Fig. 3:**
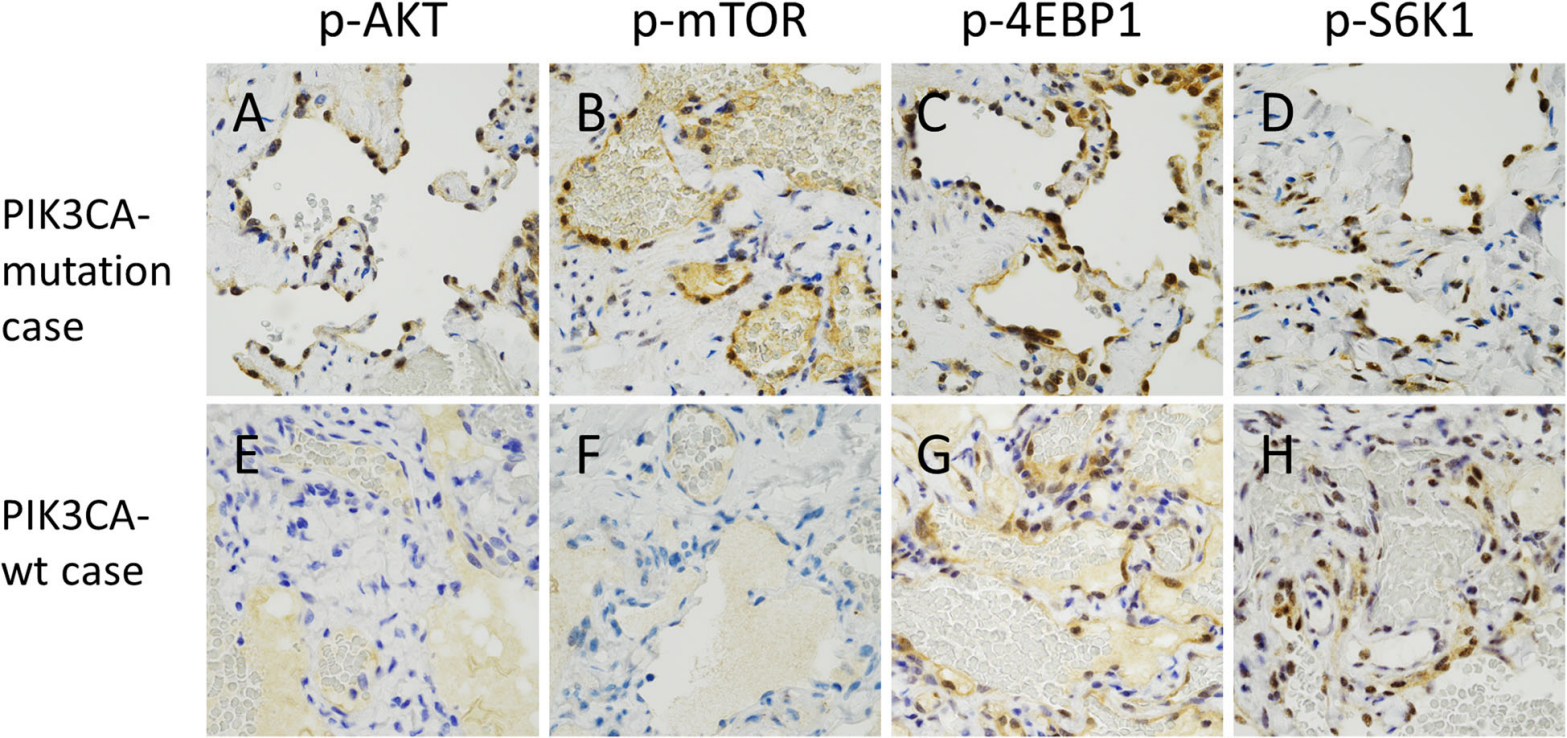
Immunohistochemical expression of the mTOR pathway in abnormal vessels of FAVA. **A-H** Representative immunohistochemical staining patterns of abnormal vessels in PIK3CA-mutation case (**A-D**) and PIK3CA-wild type (wt) case (**E-H**). Both cases express phosphorylated eukaryotic translation initiation factor 4E-binding protein 1 (p-4EBP1) and phosphorylated ribosomal protein S6 kinase 1 (p-S6K1), but only the PIK3CA-mutation case expresses phosphorylated AKT (p-AKT) and phosphorylated mammalian target of rapamycin (p-mTOR). The PIK3CA-wt case does not express p-AKT or p-mTOR

We next examined the mTOR pathway activation status in abnormal vessels using immunohistochemical staining. The expression of p-AKT and p-mTOR, both of which are upstream of the mTOR pathway, was detected in abnormal vessels of the PIK3CA-mutation cases (cases 1 and 2) (Fig. 3 A, B) but not in those of the PIK3CA-wt cases (cases 3 and 4) (Fig. 3E, F). The expression of p-4EBP1 and p-S6K1, both of which are downstream effectors of the mTOR pathway, was detected in abnormal vessels of both PIK3CA-mutaion and PIK3CA-wt cases (Fig. 3 C, D, G, H). The one PIK3CA-wt case (case 4) dose not express p-4EBP1 expression. In normal tissues, including the surrounding skeletal muscle and normal vessels, p-S6K1 exhibited sporadic expression, while p-AKT, p-mTOR, and p-4EBP1 were not expressed at detectable levels. The immunohistochemical results are summarized in Table 3.

**Table 3:** Immunohistochemical expression of mTOR pathway in abnormal vessels

Case	p-AKT	p-mTOR	p-4EBP1	p-S6K1
1	+	+	+	+
2	+	+	+	+
3	-	-	+	+
4	-	-	-	+

Staining intensity (-; no expression / +; positive)

## Discussion

FAVA is a new entity of vascular anomaly and is exceedingly rare. Alomari et al. (2014) [1] provided a proposed definition of the clinical and histological characteristics of FAVA. Subsequently, *PIK3CA* mutations were reported in a subset of FAVAs (5/8 cases) [4]. However, the correlations between specific mutations and clinicopathological features remain unclear. The current study is the first reported series of clinical, histological, immunohistochemical, and genetic analyses examining FAVA cases.

According to a clinical series of FAVA, FAVA arises in young patients (median age, 12-17 years) [1, 2]. Common symptoms include pain (100%), functional restriction (81-100%), and swelling (36.5%) [1, 2]. The most common location of the lesion is the lower extremities (94.7%) [2]. Our current study determined that the median age of PIK3CA-mutation patients was 9 years and that of PIK3CA-wt patients was 12.5 years. Both PIK3CA-mutation and PIK3CA-wt patients presented with pain, swelling, and functional restriction. All four lesions were located in the lower extremities. Based on the above findings, our cases exhibited typical clinical features regardless of *PIK3CA* mutational status (Table 1) [1, 2]. Similarly, both PIK3CA-mutation and PIK3CA-wt cases exhibited the typical histological features of FAVA (Table 2). Histologically, VM, LM, fibrous tissue, and adipose tissue were observed in all cases. Nerves containing enlarged vessels, a condition that is unusual in other vascular anomalies, were also present regardless of *PIK3CA* mutational status (Fig. 2D, H). Thus, our observations indicated that there was no significant association between the presence of *PIK3CA* mutations and the clinicopathological features of FAVA.

Immunohistochemical analysis showed that p-AKT and p-mTOR that act upstream of the mTOR pathway were detected in abnormal vessels of PIK3CA-mutation cases, but not in those of PIK3CA-wt cases (Table 3). On the other hand, p-4EBP1 and p-S6K1, downstream of the mTOR pathway, were detected in abnormal vessels of both PIK3CA-mutation and PIK3CA-wt cases (Table 3). One interpretation of this discrepancy was that 4EBP1 and S6K1 were activated in mTOR-independent manner. In fact, phosphorylation of 4EBP1 and S6K1 is subject to mTOR-independent several kinases and feedback loops [14, 15]. Somatic mutations in *PIK3CA* occur frequently in cancers other than LMs and other PIK3CA-related overgrowth spectrums [4–6, 8]. In cancers, a small number of studies have demonstrated a positive correlation between *PIK3CA* mutational status and upstream activation of the mTOR pathway [16–18]. p-AKT and p-mTOR were immunohistochemically expressed more frequently in PIK3CA-mutation cases than in PIK3CA-wt cases, while the immunohistochemical expression of p-4EBP1 and p-S6K1 was not correlated with the presence of *PIK3CA* mutation [16–18]. These results were consistent with the relationship between the mutation and immunohistochemical expression in our FAVA cases.

Both 4EBP1 and S6K1 are involved in the development of abnormal vessels in VMs and LMs by promoting protein synthesis and cell growth [5–9, 11, 12]. The activation of 4EBP1 and S6K1 may play a key role in the pathogenesis of abnormal vessels in FAVA lesions; however it was unclear what signaling pathways were involved in their activation. Since the identification of *PIK3CA* mutations in FAVA by Luks et al. [4], further mutational analyses of FAVA have not been performed. Our targeting NGS failed to identify common gene mutations associated with mTOR pathway among PIK3CA-wt cases, although *TEK* mutation (p.R842H within exon 13) was detected in one PIK3CA-wt case. Somatic gain-of-function mutations in *TEK* gene that encodes the endothelial tyrosine-protein kinase receptor TIE-2 occurs approximately half of sporadic VMs and in a subset of LMs [8, 19, 20]. *TEK* hotspot mutations are detected exclusively in exon 17 and are present within the first tyrosine kinase and kinase insert domains of the receptor [8, 19, 20]. *TEK* hotspot mutations result in a constantly active PI3K/AKT signaling pathway involving angiogenesis [8, 19, 20]. On the other hand, AKT phosphorylates many downstream molecules involved in the regulation of cellular functions. Therefore, little is known about the association with *TEK* mutations and activation of mTOR downstream effectors in VMs. The identified *TEK* p.R842H (c.2525G>A) mutation in current study is reported in the COSMIC (Catalogue of Somatic Mutations in Cancer) database, however the function of this mutation is not investigated. Considering that activated AKT was not detected in PIK3CA-wt cases, the *TEK* p.R842H mutation may not activate AKT in FAVA. Approximately 25% of VMs lacked both *TEK* and *PIK3CA* mutations [8, 10], and the responsible genetic aberrations remain unclear. Thus, FAVA lacking *PIK3CA* mutations may be caused by undiscovered mutations that activate 4EBP1 and S6K1.

## Conclusions

In this study, we reported the results of clinical, histological, immunohistochemical, and genetic analyses examining a small series of isolated FAVA. There was no significant association between the presence of *PIK3CA* mutations and the clinical and histological features of FAVA, suggesting that the *PIK3CA* gene may be not necessarily involved in the onset of FAVA. FAVA lacking *PIK3CA* mutations may be caused by other mutations that activate 4EBP1 and S6K1.

## Data Availability

The surgical materials and the datasets analyzed during the current study are available from the corresponding author on reasonable request.

## Abbreviations

4EBP1: Eukaryotic translation initiation factor 4E-binding protein
AKT1: AKT serine/threonine kinase 1
BRAF: B-Raf proto-oncogene, serine/threonine kinase
FAVA: Fibro-adipose vascular anomaly
FFPE: Formalin-fixed paraffin-embedded
GNA11: G protein subunit alpha 11
GNAQ: G protein subunit alpha q
HRAS: HRas proto-oncogene, GTPase
KRAS: KRAS proto-oncogene, GTPase
KTS: Klippel-Trenaunay syndrome
LM: Lymphatic malformation
MAP3K3: Mitogen-activated protein kinase kinase kinase 3
mTOR: Mammalian target of rapamycin
NRAS: NRAS proto-oncogene, GTPase
PCR: Polymerase chain reaction
PI3K: Phosphoinositide 3-kinase
PIK3CA: Phosphatidylinositol-4, 5-bisphosphate 3-kinase catalytic subunit alpha
PTEN: Phosphatase and tensin homolog
RASA1: RAS p21 protein activator 1
S6K1: Ribosomal protein S6 kinase 1
TEK: TEK receptor tyrosine kinase
VM: Venous malformation
WT: Wild-type
