# Correction: *PIK3CA* mutation correlates with mTOR pathway expression but not clinical and pathological features in Fibro-adipose vascular anomaly (FAVA)

**DOI:** 10.1186/s13000-022-01224-5

**Published:** 2022-05-02

**Authors:** Yumiko Hori, Katsutoshi Hirose, Michio Ozeki, Kenji Hata, Daisuke Motooka, Shinichiro Tahara, Takahiro Matsui, Masaharu Kohara, Hiroki Higashihara, Yusuke Ono, Kaishu Tanaka, Satoru Toyosawa, Eiichi Morii

**Affiliations:** 1 Department of Pathology, Osaka University Graduate School of Medicine, 2-2 Yamadaoka, 565-0871 Suita, Osaka, Japan; 2 Department of Central Laboratory and Surgical Pathology, National Hospital Organization, Osaka National Hospital, 2-1-14 Hoenzaka, Chuo-ku, Osaka-shi, Osaka, 540-0006 Japan; 3 Department of Oral Pathology, Osaka University Graduate School of Dentistry, 1-8 Yamadaoka, 565-0871 Suita, Osaka, Japan; 4 Department of Pediatrics, Graduate School of Medicine, Gifu University, 1-1 Yanagido, Gifu, 501-1194 Japan; 5 Department of Molecular and Cellular Biochemistry, Osaka University Graduate School of Dentistry, 1-8 Yamadaoka, 565-0871 Suita, Osaka, Japan; 6 Genome Information Research Center, Research Institute for Microbial Diseases, Osaka University, 3-1 Yamadaoka, 565-0871 Suita, Osaka, Japan; 7 Department of Radiology, Osaka University Graduate School of Medicine, 2-2 Yamadaoka, 565-0871 Suita, Osaka, Japan

**Correction to: Diagn Pathol 17, 19 (2022)**

**https://doi.org/10.1186/s13000-022-01199-3**

Following publication of the original article [1], the authors noticed the incorrect article title in the published article. The correct article title is “*PIK3CA* mutation correlates with mTOR pathway expression but not clinical and pathological features in Fibro-adipose vascular anomaly (FAVA)”.The original article has been updated.
